# RRD-251 enhances *all-trans* retinoic acid (RA)-induced differentiation of HL-60 myeloblastic leukemia cells

**DOI:** 10.18632/oncotarget.10136

**Published:** 2016-06-17

**Authors:** Aaron S. Wallace, Harrison T. Supnick, Rodica P. Bunaciu, Andrew Yen

**Affiliations:** ^1^ Department of Biomedical Sciences, Cornell University, Ithaca, NY 14853, USA

**Keywords:** all-trans retinoic acid (RA), RRD-251, GSK-3, c-Raf, retinoblastoma protein (RB)

## Abstract

*All-trans*-retinoic acid (RA) is known to induce terminal granulocytic differentiation and cell cycle arrest of HL-60 cells. Responding to an RA-induced cytosolic signaling machine, c-Raf translocates to the nucleus, providing propulsion for RA-induced differentiation. This novel mechanism is not understood, but presumably reflects c-Raf binding with nuclear gene regulatory proteins. RRD-251 is a small molecule that prevents the interaction of c-Raf and RB, the retinoblastoma tumor suppressor protein. The involvement of c-Raf and RB in RA-induced differentiation motivates interest in the effects of combined RA and RRD-251 treatment on leukemic cell differentiation.

We demonstrate that RRD-251 enhances RA-induced differentiation. Mechanistically, we find that nuclear translocated c-Raf associates with pS608 RB. RA causes loss of pS608 RB, where cells with hypophosphorylated S608 RB are G_0/_G_1_ restricted. Corroborating the pS608 RB hypophosphorylation, RB sequestration of E2F increased with concomitant loss of cdc6 expression, which is known to be driven by E2F. Hypophosphorylation of S608 RB releases c-Raf from RB sequestration to bind other nuclear targets. Release of c-Raf from RB sequestration results in enhanced association with GSK-3 which is phosphorylated at its S21/9 inhibitory sites. c-Raf binding to GSK-3 is associated with dissociation of GSK-3 and RARα, thereby relieving RARα of GSK-3 inhibition. RRD-251 amplifies each of these RA-induced events. Consistent with the posited enhancement of RARα transcriptional activity by RRD-251, RRD-251 increases the RARE-driven CD38 expression per cell. The RA/c-Raf/GSK-3/RARα axis emerges as a novel differentiation regulatory mechanism susceptible to RRD-251, suggesting enhancing RA-effects with RRD-251 in therapy.

## INTRODUCTION

All-trans-retinoic acid (RA) is known to induce myeloid differentiation and cell cycle arrest in HL-60 lineage bipotent acute myeloblastic cells (a non-APL, NCI-60 reference cell line). RA differentiation therapy revolutionized the treatment of acute promyelocytic leukemia (APL), a rare subtype of myeloid leukemia. From once being considered one of the most difficult to medically manage, APL is now one of the most treatable, with cure rates of 80-90% using combined RA and arsenic trioxide treatment [1]. The resounding success of RA treatment with APL, the lower toxicity of RA compared to traditional chemotherapeutic agents, and the lack of secondary tumors emerging much later as a sequela has motivated immense interest in its use in other cancers and leukemias, especially in acute myeloid leukemia (AML), where there is uncontrolled proliferation of non-terminally differentiated myeloid precursor cells [2, 3]. Currently, chemotherapy for AML can achieve remission in 60 to 80% of patients less than 60 years of age [4]. Most, however, relapse with cancers that are treatment-resistant within 2-3 years, and 5-year survival rates are as low as 30% [5, 6]. Unfortunately, treatment with RA does not induce differentiation in AML, only apoptosis or inhibition of proliferation [7]. Recent studies have revealed that inhibitors of glycogen synthase kinase-3 (GSK-3) can induce differentiation in AML cells and confer RA-susceptibility by activating RARα transcriptional activity [8-10]. GSK-3 inhibitors also demonstrate acceptable toxicity [11]. Thus, there is both a critical need and opportunity to expand and broaden the anti-cancer properties of RA using combination therapies.

In HL-60 cells, a durable signal through the MAPK pathway drives differentiation, where c-Raf (MAPKKK) acts as a vital signaling member [12-14]. The traditional MAPK pathway holds that c-Raf phosphorylates MEK, MEK phosphorylates ERK, and ERK phosphorylates and regulates transcription factors in the nucleus, following its nuclear translocation [15]. In this historical paradigm, c-Raf acts as a transducer of membrane receptor signals that is recruited to the plasma membrane to phosphorylate MEK, which was thought to be one of c-Raf's few targets [16, 17]. Surprisingly, following RA treatment, c-Raf is phosphorylated at serine 621 and is translocated to the nucleus by 48 hours [18]. In the nucleus, c-Raf targets and activates transcription factors, in particular NFATc3, which promotes transcription of genes necessary for differentiation; and inhibition of the c-Raf-NFATc3 interaction by PD98059 inhibits differentiation [19]. Overexpression of c-Raf enhances differentiation through augmented MAPK and BLR1 signaling, while its inhibition attenuates differentiation [12]. These results indicate that c-Raf has targets additional to MEK and plays a pivotal role in driving differentiation. This novel nuclear action of c-Raf with RA treatment motivates interest in its targets and may illuminate the mechanism by which RA changes mediators of proliferation and transformation to inducers of arrest and differentiation.

Retinoblastoma tumor suppressor protein (RB) is a key regulator of the cell cycle in HL-60 cells. For HL-60 cells, G_1_/S/G_2_ progression is associated with progressively greater RB hyperphosphorylation, while hypophosphorylated RB is only detectable after RA treatment, most significantly by 72 hours as cells G_0_ arrest [20, 21]. Hypophosphorylated RB ostensibly sequesters E2F transcription factors, preventing their activation of dependent promoters at genes whose products are required for entrance into S phase [22]. In RA-induced differentiation, the formation of RB-E2F complexes appeared at 48 hours after treatment [23]. Phosphorylation of RB at serine 608 induces a conformational change that prevents the binding of RB to the E2F family of transcription factors [24]. RB is also phosphorylated by c-Raf, and previous studies showed that if the c-Raf-RB interaction early in G_1_ phase is disrupted then RB could not be hyperphosphorylated, even in late G_1_ phase [25, 26]. These cells then could not enter S-phase, which implies that RB phosphorylation by c-Raf is essential for the hyperphosphorylation of RB. Hence, if RB phosphorylation by c-Raf is indeed necessary for cell cycle progression, then blocking this interaction could be exploited to induce cell cycle arrest.

Chellappan and colleagues synthesized a small molecule, RRD-251, that selectively inhibited the binding of c-Raf to RB [27]. By inhibiting this interaction, RRD-251 prevented the hyperphosphorylation of RB and subsequently inhibited E2F driven transcriptional activity [22]. The ability of RRD-251 to inhibit cellular proliferation was directly linked to the presence of functional RB. In cancer cells without functional RB, such as DU145 prostate cancer cells, RRD-251 had no effect on proliferation [27]. Interestingly, RRD-251 also inhibits angiogenesis in vivo and in vitro and leads to tumor size reduction [27].

Here, we report that RRD-251 enhances RA-induced differentiation, measured by cell cycle arrest, expression of the differentiation cell surface marker CD11b, and the functional marker respiratory burst/inducible oxidative metabolism. Seeking mechanistic insight, we find that RA induces a transient nuclear association of c-Raf and hyperphosphorylated pS608 RB. When c-Raf is freed from pS608 RB sequestration by pS608 RB hypophosphorylation in RA-treated cells, where cells with hypophospshorylated pS608 RB are G_0/_G_1_ restricted; it binds GSK-3 which is phosphorylated at the inhibitory phosphorylation site, serine 9/21, which is known to suppress the activating phosphorylation of GSK-3 at tyrosine 216/279. By dissociating c-Raf from pS608 RB, RRD-251 enhances this process. Hence RA-driven modulations in GSK-3 phosphorylation are significantly augmented in RA plus RRD-251 co-treated cells. RA thus induces a novel interaction between c-Raf and GSK-3 which is enhanced by RRD-251. At the same time RA and RRD-251 also inhibit the association between GSK-3 and ERK. Most significantly, RA and even more so when with RRD-251 inhibits the GSK-3-RARα interaction. GSK-3 is known to diminish RARα transcriptional activity, so reducing the interaction relieves the inhibition of RARα by GSK-3. While the effect of RA alone on these interactions is modest, the amplification of RA activity on these interactions by RRD-251 is striking. As evidence corroborating enhanced RARα transcriptional activity, we observe an increased RA-induced CD38 expression per cell in co-treated samples; CD38 expression is driven by an RARE, making it a reporter of RA-induced transcriptional activation via RARα. Our results indicate a novel RA/c-Raf/GSK-3/RARα axis whereby RA enhances RARα driven transcriptional activity by mitigating an inhibitor, namely GSK-3, and cell differentiation is enhanced. The RA/c-Raf/GSK-3/RARα axis thus emerges as a novel differentiation regulatory mechanism susceptible to RRD-251 regulation with enhanced blast differentiation as the end result. This suggests the potential use of combined therapy with RRD-251 in AML cells.

## RESULTS

### RRD-251 enhances RA-induced differentiation

To determine if RRD-251 affected RA-induced differentiation, HL-60 cells were treated with each drug alone or in combination. RA-induced granulocytic differentiation is characterized by growth arrest and expression of several phenotypic differentiation markers. These include: the surface receptors CD38, CD11b, and inducible respiratory burst - a functional marker of fully matured neutrophils. By itself, RRD-251 treatment induces cell cycle arrest, but in combination with RA it enhances the expression of each of these markers as compared to RA alone.

**Figure 1:**
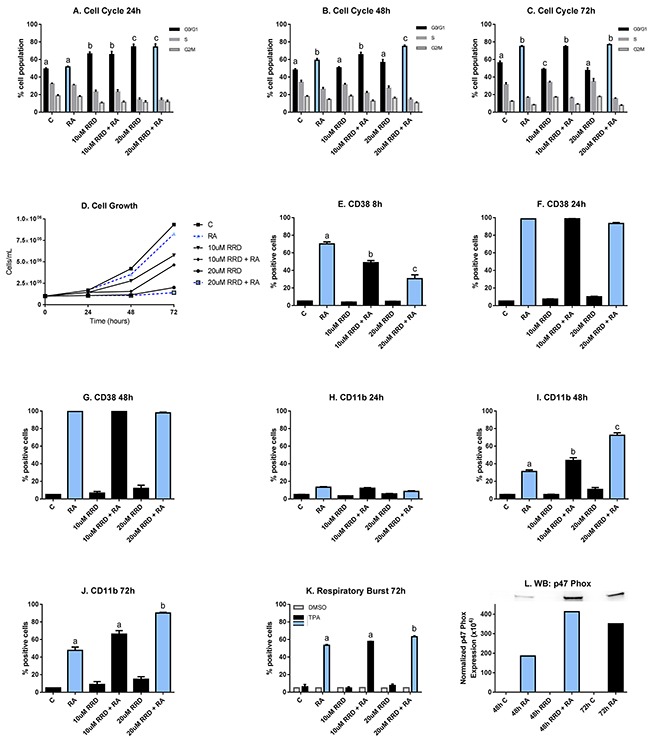
RRD-251 enhances RA-induced differentiation HL-60 cells were untreated (C) or treated with 1 μM RA, 10 or 20 μM RRD-251, or RA plus 10 or 20 μM RRD-251 for the designated time points. The statistically significant changes are labeled a, b, c on the graphs. **A.** Cell cycle distribution (measured by flow cytometry with propidium iodide staining) at 24 hours after treatment indicates a dose-dependent enrichment of G_0_/G_1_ by RRD-251(C vs. 10 μM RRD-251 p<0.05; C vs. 20 μM RRD-251 p<0.001). **B.** At 48 hours post treatment, RA induces cell cycle arrest (p<0.0002) and cells treated with both 20 μM RRD-251 and RA exhibited even greater levels of cell cycle arrest than RA alone (p<0.005). **C.** By 72 hours after treatment, enrichment in G_0_/G_1_ is solely dependent on RA treatment (p<0.01). **D.** Cell density was measured for both treated and untreated cells at 0, 24, 48, and 72 hours. RRD-251 alone significantly inhibited growth in a dose dependent manner and enhances RA-induced growth inhibition. **E.** CD38 expression (assessed by flow cytometry with a PE-conjugated antibody) at 8 hours post treatment is induced by RA, but inhibited in dose dependent manner by RRD-251 (RA vs 10 μM RRD-251 + RA p=0.001; 10 μM RRD-251 + RA vs 20 μM RRD-251 p<0.05). **F.** At 24 hours post treatment, CD38 expression by RA is nearly 100% and is not inhibited by RRD-251 treatment. **G.** At 48 hours post treatment, CD38 expression by RA is nearly 100% and is not inhibited by RRD-251 treatment. **H.** CD11b expression (assessed by flow cytometry with an APC-conjugated antibody) at 24 hours post treatment is slightly induced by RA with no affect with RRD-251 treatment. **I.** At 48 hours, RA-induced expression of CD11b increases and is augmented with RRD-251 treatment (RA vs 10 μM RRD-251 + RA p<<0.05; 10 μM RRD-251 + RA vs 20 μM RRD-251 p<0.0005). **J.** At 72 hours, RRD-251 again enhances RA-induced expression of CD11b (RA vs 20 μM RRD-251 + RA p<0.05). **K.** Respiratory burst, measured by flow cytometry of DCF stained cells, is significantly enhanced at 72 hours by co-treatment compared to RA alone (p=0.021). **L.** Expression of p47^phox^ was measured by Western blotting at 48 hours for control, RA, 20 μM RRD-251, and RA+ 20 μM RRD-251 and at 72 hours for control and RA. RRD-251 significantly augments the RA-induced expression, where there is greater expression in the RRD-251 plus RA samples at 48 hours than the RA samples at 72 hours. All the experiments were performed three times (three biological repeats).

HL-60 cells were untreated (C) or treated with RA, 10 or 20 μM RRD-251, or RA plus 10 or 20 μM RRD-251. Cell population growth was assessed by cell counting using a hemocytometer. RA achieves remission through the maturation of non-terminally differentiated myeloblasts into granulocytes, which lose the ability to proliferate and self-renew. Thus, a marker of differentiation is cell cycle arrest in G_0/_G_1_. The cell cycle distribution was analyzed for samples at 24, 48, and 72 hours after treatment with both drugs (Figure 1A-1C). At 24 hours after treatment, RRD-251 induces cell cycle arrest in a dose dependent manner (C vs. 10 μM RRD-251 p<0.05; C vs. 20 μM RRD-251 p<0.001) (Figure 1A). At 48 hours after treatment, RA induces cell cycle arrest (p<0.0002). Cells treated with both 20μM RRD-251 and RA exhibit even greater levels of cell cycle arrest than RA alone (p<0.005) (Figure 1B). By 72 hours after treatment, enrichment in G_0/_G_1_ is solely dependent on RA (p<0.01) (Figure 1C). So RRD-251 enhances RA-induced cell cycle arrest in G_0/_G_1._

Growth curves were consistent with changes in the cell cycle phase distribution (Figure 1D). RRD-251 alone significantly inhibited cellular growth (0.25 × 10^6^ cells/mL for 20 μM RRD-251 and 0.9 × 10^6^ cell/mL for untreated cells at 72 h after treatment). RRD-251 treatment with RA further lowered the cell densities compared to both RRD-251 and RA alone (0.9 × 10^6^ cells/mL for RA alone; 0.25 × 10^6^ cells/mL for 20 μM RRD-251 alone; 0.20 × 10^6^ cells/mL for combined treatment at 72 h post-treatment). The decreased population growth corroborates increased arrest in G_0/_G_1_. And both are consistent with enhanced RB hypophosphorylation to be described below.

CD38 is an ectoenzyme receptor, whose expression is induced by RA and modulates the cellular response to RA [28]. Expression of CD38 is an early indicator of differentiation. At 8 hours after treatment, RA induces the expression of CD38, which is delayed by RRD-251 in a dose-dependent manner (RA vs 10 μM RRD-251 + RA p=0.001; 10 μM RRD-251 + RA vs 20 μM RRD-251 p<0.05) (Figure 1E). At 24 hours and 48 hours after RA treatment, the percentage of cells expressing CD38 is nearly 100% and is not inhibited by RRD-251 treatment (Figure 1F-1G). RRD-251 thus briefly dampens RA-induced CD38 expression, an effect that is subsequently lost.

CD11b expression is a later marker of differentiation. It is the integrin α M subunit of heterodimeric integrin (α_M_β_2_), also known as complement receptor 3 and is typically associated with the activation and maturation of innate immune cells such as granulocytes and monocytes [29-31]. At 24 hours after treatment, RA just begins inducing CD11b expression and there is no effect of RRD-251 treatment yet (Figure 1H). At 48 hours, RA-induced CD11b expression increases and is augmented by RRD-251 co-treatment (RA vs 10 μM RRD-251 + RA p<0.05; 10μM RRD-251 + RA vs 20μM RRD-251 p<0.0005) (Figure 1I). At 72 hours, RA-induced expression of CD11b continues being enhanced by RRD-251 (RA vs 20 μM RRD-251 + RA p<0.05) (Figure 1J). RRD-251 thus enhances RA-induced CD11b expression.

The production of reactive oxygen species (ROS) is a functional marker of neutrophils, and it is produced by the NADPH oxidase complex to kill phagocytized bacteria [32]. This late differentiation marker for functionally mature differentiated cells is not strongly apparent until 72 hours after RA treatment. At that time, nearly 50% of RA-treated cells are positive for ROS production, while cells treated with 20 μM RRD-251 and RA have over 60% of cells positive (p<0.05) (Figure 1K). An associated cytosolic marker of differentiation is the expression of p47^phox^, a subunit of the NADPH oxidase complex producing the respiratory burst. RA-induced p47^phox^ expression is enhanced by addition of RRD-251. Indeed, expression in cells treated with RA plus RRD-215 for 48 hours exceeds that in cells treated with just RA for 72 hours, indicating a significant acceleration in the differentiation process (Figure 1L). RRD-251 thus enhances RA-induced differentiation measured by CD11b expression, inducible oxidative metabolism, and G_0/_G_1_ arrest.

RRD-251 is a known disruptor of the c-Raf kinase-RB protein interaction, motivating interest in the molecular basis of how this might have promoted RA-induced differentiation. The mobilization of c-Raf to the nucleus following RA treatment propels differentiation [18]. This suggested an initial conjecture that c-Raf targeted various nuclear molecules and that dissociation from RB induced by RRD-251 may favor others with the effect of promoting differentiation. A number of candidate mechanisms amongst the putative mediators of RA-induced differentiation were evaluated as potential c-Raf targets. These included RB hyper/hypophosphorylation, cell cycle regulatory cyclin and E2F related events, and transcription factor activation.

### RA causes a pS608 RB - c-Raf interaction disrupted by RRD-251

**Figure 2:**
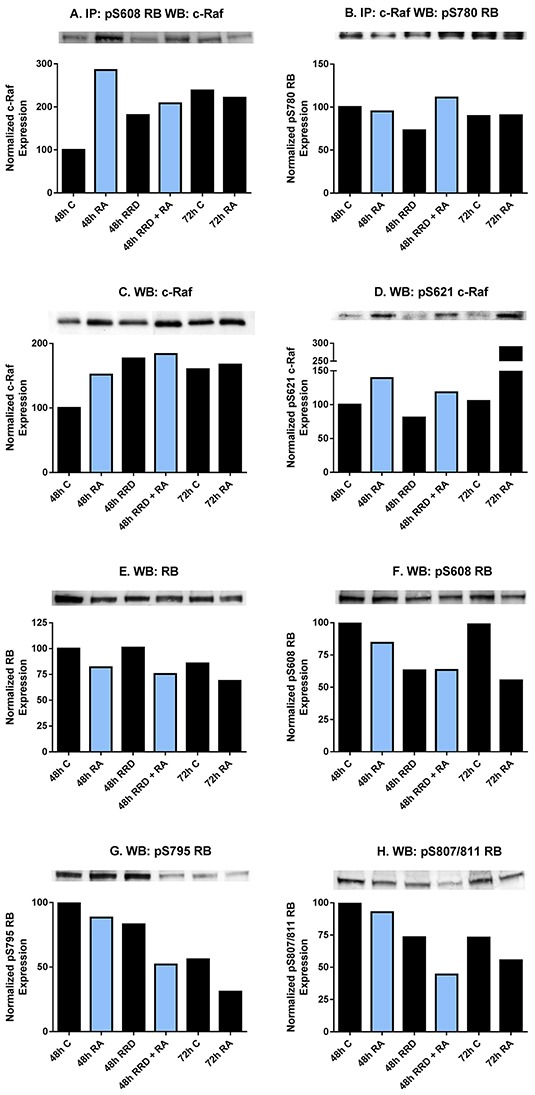
RA and RRD-251 promote transient changes in the c-Raf-RB interaction and their specific phosphorylation 300 μg of pre-cleared nuclear lysate collected 48 or 72 hours post treatment were incubated overnight with 1 μg of the precipitating antibody with magnetic beads and resolved on 7.5% polyacrylamide gels. **A.** A pS608 RB antibody was used as the precipitating antibody and the blot was probed by overnight incubation with c-Raf primary antibody. RA promotes a transient nuclear interaction between c-Raf and pS608 RB at 48, but not at 72 hours post treatment. The presence of RRD-251 disrupts the RA-promoted interaction at 48 hours, where co-treated samples at 48hours post treatment resemble 72 hours 1 μM RA-treated samples. **B.** A c-Raf antibody was used as the precipitating antibody and the blot was probed with pS780 RB primary antibody. There is not any significant modulation in the interaction between c-Raf and pS780 RB with either drug treatment. **C.** Changes in the specific phosphorylation of c-Raf and RB were assessed by Western blotting. Nuclear lysates collected 48 or 72 hours after treatment were resolved on 7.5% polyacrylamide gels. RRD-251 samples were only collected 48 hours after treatment. 25 μg of protein was loaded per well. RA induces the nuclear translocation of c-Raf, which is augmented by RRD-251 treatment. **D.** RA promotes the phosphorylation of c-Raf at serine 621 at 48 and 72 hours after treatment, which is not enhanced in co-treated samples. **E.** RA promotes the downregulation of total RB. **F.** RA promotes a decrease in pS608 RB levels at 48 and 72 hours, but most significantly at 72 hours. This decrease is amplified with RRD-251 treatment. **G-H.** Westerns of pS795 RB and pS807/811 RB indicate that RA induces the global dephosphorylation of RB at 72 hours, which is observed in co-treated samples at 48 hours post treatment.

To determine functions for c-Raf in the nucleus, its interaction with potential partners was analyzed. RA-induced nuclear translocated c-Raf associates with pS608 RB, evident at 48 hours post-treatment (Figure 2A). The enhanced association in RA-treated cells compared to untreated controls was diminished by adding RRD-251. RA-enhanced association compared to control was no longer evident at 72 hours when association in control cells also increased, possibly reflecting the high cell density in control cells. In contrast to pS608 RB, the association of c-Raf and pS780 RB did not change this way with either drug treatment or time, and was relatively stable (Figure 2B). The RA-induced enhanced association of c-Raf and pS608 RB occurs with increased levels of c-Raf and specifically pS621 c-Raf in the nucleus and is not attributable to any increase in RB protein or pS608 RB in the nucleus (Figure 2C-2F). RA induces the nuclear translocation of c-Raf and its phosphorylation at serine 621. RRD-251 by itself interestingly also increases the total amount of nuclear c-Raf, but not the phosphorylation at serine 621 (Figure 2C-2D). RA promotes the downregulation of total RB (Figure 2E). While increasing the amount of complexed c-Raf and pS608 RB, RA diminishes the amount of pS608 RB (Figure 2F). Serine 608 is a critical residue whose phosphorylation induces a conformational change in RB preventing its binding to E2F transcription factors [24]. By 48 hours RRD-251 reduces pS608 RB to levels similar to 72 hours RA-treated samples, ergo accelerating the process of pS608 hypophosphorylation and reducing the amount of RB available to sequester c-Raf. RA also induces the global dephosphorylation of RB [20, 33]. The dephosphorylations at S608, 795, 807/811 present in RA/RRD-251 co-treated cells at 48 hours post treatment compares to that at 72 hours for RA alone, indicating an acceleration of the dephosphorylation process (Figure 2F-2H). Taken together we see that RA causes the amount of c-Raf – pS608 RB complex to increase, and this is associated with increased c-Raf in the nucleus but not increased pS608 RB, which is in fact decreasing with RA. RRD-251 diminishes the amount of complexed c-Raf - pS608 RB while also reducing the amount of pS608 RB, and so the increased nuclear c-Raf is freed from RB sequestration to go to other potential targets.

### RRD-251 enhances pS608 RB hypophosphorylation

Serine 608 RB is of significance because it is the site where phosphorylation dissociates E2F from RB to drive S phase. It is also the site in the present context where dephosphorylation is associated with the divorce of c-Raf from RB. Immunofluorescent staining for pS608 RB and flow cytometry were used to analyze pS608 hypo- versus hyper- phosphorylated RB in cells treated with RA and RRD-251. We observe that changes in pS608 RB are quantized, namely that there are discrete cell subpopulations with hyper- or hypo-phosphorylated serine 608 RB and cells with the hypo phosphorylated are restricted to G_0/_G_1_, and that RRD-251 induces pS608 RB hypophosphorylation.

**Figure 3:**
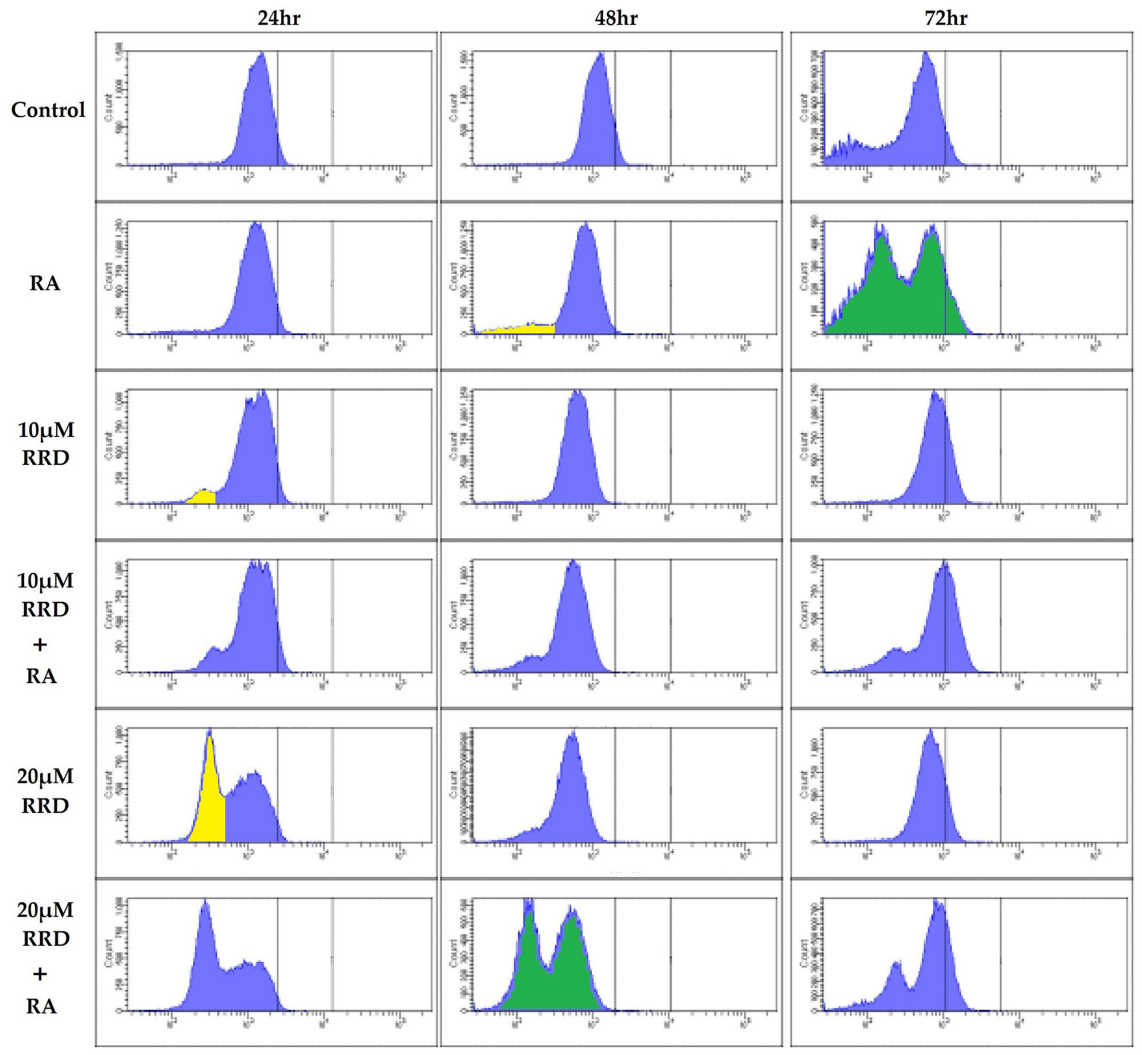
RB serine 608 phosphorylation is modulated by RA and RRD-251 Cells were collected at 24, 48, and 72 hours after experiment initiation and fixed for 7 minutes in 100 μL 2% para-formaldehyde PBS solution and permeabilized with 900 μL ice-cold methanol. Following several washings with PBS, samples were incubated with pS608 RB primary antibody overnight. The samples were incubated with a FITC goat anti-rabbit secondary antibody and analyzed by flow cytometry. Cells were either control (Row 1), 1 μM RA (Row 2), 10 μMRRD-251 (Row 3), 10 μMRRD-251+1 μM RA (Row 4), 20 μMRRD-251 (Row 5), or 20 μMRRD-251+ 1 μM RA (Row 6) and collected at 24, 48, and 72 hours. At 24 hours, RRD-251 induces a population with hypophosphorylated serine 608 RB. At 48 hours, the population is present in RA and RRD-251 samples. RA induces the population at 72 hours. Comparing the two green histograms shows that RA induces a hypo and hyper pS608 RB by 72 hours whereas RA+RRD-251 does it by 48 hours. The yellow marks hypo phosphorylated pS608 RB. Three biological repeats were analyzed.

Immunofluorescent staining for pS608 RB and flow cytometry reveal a lower hypo- and higher hyper- phosphorylated pS608 RB subpopulations. Interestingly the discrete peaks show that for any given cell its RB is either essentially all in the hypo (lower peak) or in the hyper (upper peak) phosphorylated state at serine 608 – reminiscent of an on/off binary states such as the negative and positive subpopulations for cell surface markers of leukocytes (Figure 3). Hypophosphorylated S608 RB is first apparent in RA treated cells at 48 hours and becomes prominent at 72 hours. Adding RRD-251 makes the hypophosphorylated S608 RB even more prominent earlier, namely by 24 hours (yellow peak, Figure 3). Hence RRD-251 hastens the occurrence of the hypophosphorylation of S608 RB seen in RA treated cells. As shown above, this would result in increased c-Raf freed from RB. The enrichment in the hypophosphorylated S608 RB population temporally corresponds with the induction of cell cycle arrest by RRD-251 at 24 hours and RA at 48 and 72 hours (Figure 1A, 1B, 1C).

### RA-induced pS608 RB hypophosphorylation is restricted to G_0/_G_1_of the cell cycle

**Figure 4:**
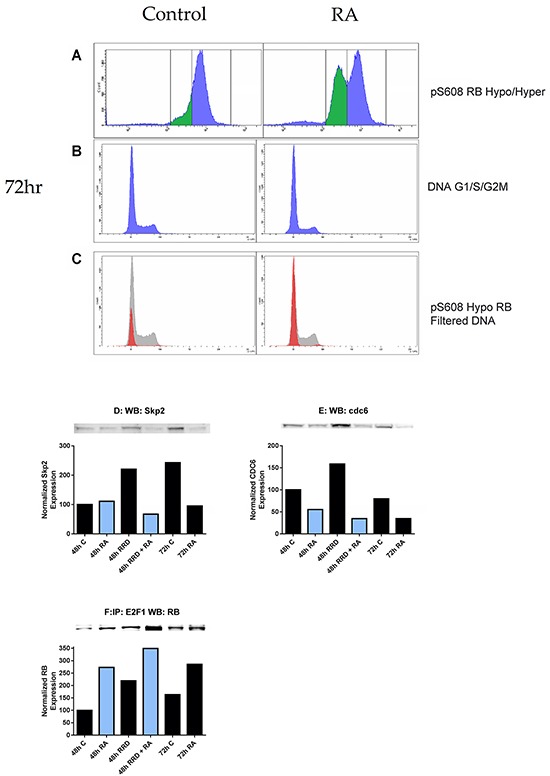
RB serine 608 phosphorylation corresponds to G_0_/G_1_ Cells were fixed and permeabilized in the same method as before, but incubated in hypotonic PI solution following the removal of the methanol/para-formaldehyde solution. Compensation of 2.0% was used to correct for spillover of PI fluorescence into the FITC channel. **A.** 1 μM RA induces the formation of a population with hypophosphorylated serine 608 RB at 72 hours. **B.** The DNA histogram (cell cycle distribution) of control and RA cells at 72 hours is depicted. **C.** The hypophosphorylated population is overwhelmingly in G_0_/G_1_ (red histogram) as compared to the hyperphosphorylated serine 608 RB population (grey histogram). **D-E.** Changes in expression of Skp2 and cdc6 were measured by Western blotting. Nuclear lysates collected 48 or 72 hours after treatment were resolved on 7.5% polyacrylamide gels. 25 μg of protein was loaded per well. **D.** RA promotes a decrease in nuclear Skp2 at 72 hours, which is observed in co-treated samples at 48 hours. **E.** RA promotes a decrease in nuclear cdc6 at both 48 and 72 hours. Levels of cdc6 are lowest in co-treated samples at 48 hours. **F.** The RB-E2F1 interaction was assessed by immunoprecipitation. 300 μg of pre-cleared nuclear lysate collected 48 or 72 hours post treatment were incubated overnight with 1 μg of the precipitating antibody with magnetic beads and resolved on 7.5% polyacrylamide gels. RA promotes the interaction between RB and E2F1 at both 48 and 72 hours after treatment as compared to control. RRD-251 alone induces this interaction and there is an additive effect with co-treatment of RA at 48 hours. Co-treated samples at 48 hours exhibit higher levels of the interaction than 72 hours RA-treated cells.

Dual immunofluorescent pS608 RB and propidium iodide DNA staining analyzed by flow cytometry were used to characterize the cellular pS608 hypophosphorylation and cell cycle. The DNA histogram of the hypophosphorylation population is over 99% in G_0/_G_1_ (Figure 4A-4C). Hence promoting hypophosphorylation of S608 RB promotes arrest. To further characterize the arrest, the effect of the RA and RRD-251 on cell cycle regulatory proteins (Skp2 and cdc6) was examined (Figure 4D-4E). SCF-Skp2 is an E3 ubiquitin ligase that targets p27 and leads to p27 instability and degradation; and RA promotes the degradation of Skp2 by the proteasome, which if disrupted inhibits differentiation [34-36]. After 72 hours when cells are differentiated, RA treatment results in decreased Skp2 levels compared to untreated control as expected for arresting cells, and addition of RRD-251 makes a comparable reduction apparent earlier, namely by 48 hours (Figure 4D). An indirect test for RB activity is the expression of E2F-driven genes. One such gene product is cdc6, which forms a critical component of the origin of replication for S phase DNA replication. RA diminishes cdc6 expression which is further diminished by adding RRD-251 consistent with enhancing arrest (Figure 4E). E2F is a cell cycle driver, and the anticipation that RA causes its sequestration at RB which is enhanced by RRD-251 was confirmed. Activated hypophosphorylated RB binds to E2F1. RA promotes this interaction and adding RRD-251 enhances it (Figure 4F). Consistent with RRD-251 alone causing loss of pS608 RB (Figure 2F), RRD-251 alone induces E2F/RB association. Hence, consistent with the shifts of hypo- and hyper- phosphorylated S608 RB shown by immunofluorescence and flow cytometry, RRD-251 generally enhanced RA-induced changes in cell cycle regulatory proteins, consistent with promoting cell cycle arrest. An inference of these findings is that the release of c-Raf from pS608 RB sequestration in RA-treated cells is cell cycle phase specific, namely in G_0/_G_1_.

### c-Raf partners in the nucleus

**Figure 5:**
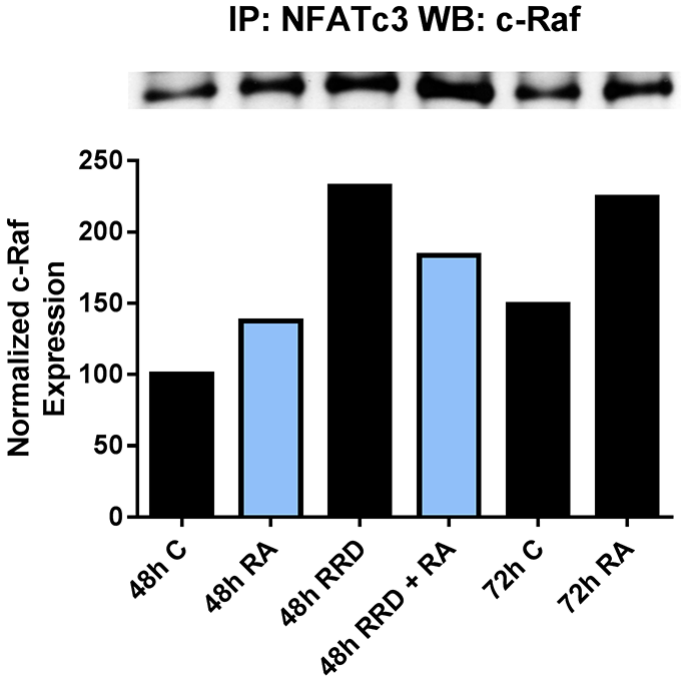
RRD-251 increases the RA-induced c-Raf-NFATc3 interaction The c-Raf-NFATc3 interaction was assessed by immunoprecipitation. 300 μg of pre-cleared nuclear lysate collected 48 or 72 hours post treatment were incubated overnight with 1 μg of the precipitating antibody with magnetic beads and resolved on 7.5% polyacrylamide gels. RA induces the c-Raf-NFATc3 interaction at 48 and 72 hours post treatment, with greater binding at 72 hours. Adding RRD-251 causes greater binding at 48 hours compared to RA-alone.

Given that an inhibitor of the c-Raf-RB interaction enhances/accelerates differentiation, we conjectured that the dissociation of c-Raf from RB by RRD-251 makes more c-Raf available to target nuclear molecules that can promote differentiation. RA increases levels of c-Raf in the nucleus, some of which becomes sequestered by pS608 RB and is then released from RB as pS608 RB is lost with RA-induced cell cycle arrest, thereby making more nuclear c-Raf available. RRD-251 dissociates c-Raf from RB and enhances this process. Hence RA causes increased nuclear c-Raf and the addition of RRD-251 further increases it (Figure 2C). RRD-251 might thus enhance c-Raf binding and activation of transcription factors to transcriptionally drive genes that propel differentiation. NFATc3 is a transcription factor that must bind and be activated by c-Raf to allow an upstream noncanonical RARE on the same promoter to activate transcription of CXCR5. [19]. CXCR5 is a gene that must be up-regulated early in the RA-induced differentiation program in order for cells to differentiate. We find that RRD-251 enhances the association of c-Raf with NFATc3 (Figure 5). RRD-251 thus enhances RA-induced c-Raf association with a transcription factor that helps drive differentiation. We evaluated other transcription factors as potential c-Raf targets, including Sp1, IRF-1, and RXRα, but none of these interactions were modified by RRD-251 (data not shown).

### RRD-251 augments RA regulation of GSK-3 activity and interactions

**Figure 6:**
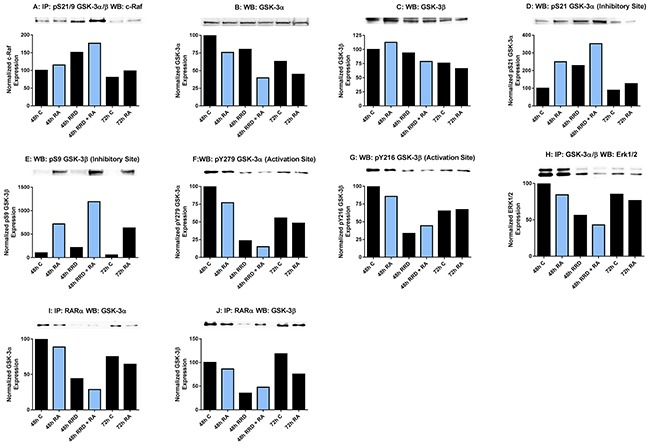
RRD-251 augments RA regulation of GSK-3 activity and interactions GSK-3 phosphorylation was assessed by western blotting and its interactions with c-Raf and RARα were assessed by immunoprecipitation. Nuclear lysates collected 48 or 72 hours after treatment were resolved on 7.5% polyacrylamide gels. RRD-251 samples were only collected 48 hours after treatment. 25 μg of protein was loaded per well for western blotting. For immunoprecipitation, 300 μg of pre-cleared nuclear lysate collected 48 or 72 hours post treatment were incubated overnight with 1 μg of the precipitating antibody with magnetic beads and resolved on 7.5% polyacrylamide gels. **A.** Immunoprecipitation of pS21/9 GSK-3α/β probed for c-Raf. RA induces a novel interaction between c-Raf and GSK-3 apparent at 48 and 72 hours. **B.** Nuclear GSK-3α levels were assessed by western blotting. Decrease in expression induced by RA is augmented by RRD-251 co-treatment. **C.** Nuclear GSK-3β levels were assessed by western blotting. Expression in RA-treated cells is decreased by adding RRD-251, as was for GSK-3α. **D-G.** Western blots of GSK-3α and β phosphorylated at inhibitory pS21/9 and activating pY279/216 sites. At 48 and 72 hours post treatment, RA induces the inhibitory phosphorylation of serine 21 and serine 9 of GSK-3. RA also slightly inhibits the activating phosphorylation of GSK-3 at tyr279 and tyr216. Addition of RRD-251 enhanced these effects. **H.** Immunoprecipitation of GSK-3α/β probed for ERK1/2. RRD-251 further reduces the diminished interaction between GSK-3 and ERK1/2 in RA-treated cells. **I-J.** Immunoprecipitation of RARα probed for GSK-3α and β RRD-251 further diminishes the GSK-3-RARα interaction in RA-treated cells, which is known to inhibit RARα transcriptional activity. The addition of RRD-251 significantly further reduces this interaction. GSK-3 is known to bind and inhibit RARα. Three biological repeats were performed and the trend for changes in expression levels are consistent among the repeats.

Seeking other partners for c-Raf in the nucleus, we found that c-Raf targets Glycogen Synthase Kinase 3 (GSK-3) and that RRD-251 causes an increase in this interaction with the overall consequence of enhancing RARα activation by inhibiting GSK-3. GSK-3 is serine threonine kinase that phosphorylates a wide range of cellular proteins and has both oncogenic and tumor suppressor activities; in particular it inhibits RARα transcriptional activity [8]. We find a novel association between nuclear c-Raf and GSK-3 which is phosphorylated at its S21/9 inhibitory sites. The interaction induced by RA is greatly increased by addition of RRD-251 (Figure 6A). This is consistent with the increase in nuclear c-Raf due to RRD-251 that was also apparent at this time after 48 hours of treatment. The effect is not attributable to changes in expression levels of the partners. GSK-3α is downregulated in RA/RRD-251 co-treated cells at 48 hours. By itself RA also causes downregulation of GSK-3α (Figure 6B). Expression of GSK-3β is also downregulated by RA/RRD-251 co-treatment (Figure 6C). Paralleling c-Raf association with GSK-3, the phosphorylation of GSK-3 at serine residues 21 (α) and 9 (β), which are inhibitory, are increased by RA and more so by RA plus RRD-251 (Figure 6D, 6E). At the same time activating phosphorylations of tyrosine residues 279 (α) and 216 (β) decrease (Figure 6F, 6G). GSK-3 is known to be regulated by the MAPK pathway, historically through phosphorylation by ERK, a priming event for GSK-3 [37]. We observe that increased c-Raf - GSK-3 binding is associated with diminished nuclear GSK-3 - ERK interaction (Figure 6H). Taken together, RRD-251 enhances the association of c-Raf with GSK-3 and phosphorylation of GSK-3 at its inhibitory site with concomitant loss of phosphorylation at the activating site.

Pursuing consequences of GSK-3 inhibition, RARα emerged as a target. GSK-3β can phosphorylate RARα, decreasing its transcriptional activity [8, 10]. Indicating a functional role for GSK-3 in differentiation, inhibitors of GSK-3 have been reported to induce granulocytic differentiation in HL-60, AML cell lines, and AML primary cells in the absence of RA, as well as enhancing RA-induced differentiation. We now find that RRD-251 greatly enhances the dissociation of both GSK-3α and β from RARα in cells treated with RA. Consistent with the ability of RRD-251 alone to increase nuclear c-Raf, RRD-251 alone also diminished the association of GSK-3 with RARα. Interestingly the dissociations of GSK-3α and β from RARα suggest the possibility that RARα regulation by GSK-3 is not limited to just the β isoform (Figure 6I-6J). RRD-251 thus promotes the dissociation of RARα from GSK-3, relieving RARα from the putative inhibitory interaction.

### RRD-251 enhances CD38 expression per cell

**Figure 7:**
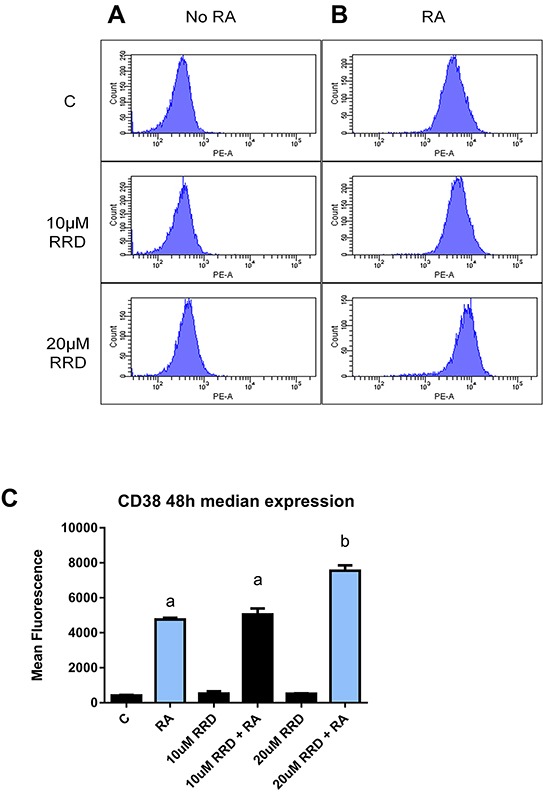
RRD-251 enhances median CD38 expression Median expression of CD38 per cell was assessed by flow cytometry using a PE-conjugated antibody) at 48 hours in control, RA-treated and RA plus RRD-251- treated cells. **A.** Histograms of CD38 expression in cells without RA show negative peak. **B.** Histograms of CD38 expression in samples treated with RA show a dose dependent increase in median expression per cell with the addition of RRD-251. (The histograms top, middle, bottom are 1 μM RA, 10 μM RRD-251+1 μM RA, 20 μM RRD-251+1 μM RA). **C.** Bar graph of median CD38 expression per cell showing that addition of RRD-251 to RA treated cells significantly increases expression (p=0.0041). Groups with different letters (a or b) are significantly different; the cells treated with 20 μMRRD-251+ 1 μM RA have significantly more CD38 than cells treated with just RA.

The GSK-3-RARα interaction is a known inhibitor of RARα. To determine if RARα transcriptional activity was enhanced by RRD-251, the median expression of the CD38 membrane protein was measured by flow cytometry. CD38 expression is driven by an RARE, making it a reporter of RA-induced transcriptional activation via RARα [38]. Cells were untreated or treated with RA or RA plus RRD-251. RRD-251 increased the amount of CD38 expressed per cell in RA-treated cells (Figure 7). There is a significant difference in the median expression of CD38 per cell (p=0.0041) between RA and 20 μM RRD-251+RA. This is consistent with the anticipation of increased transcription driven by RARα. The findings confirm the novel action of c-Raf in regulating RARα through the inhibition of GSK-3α and β.

## DISCUSSION

**Figure 8:**
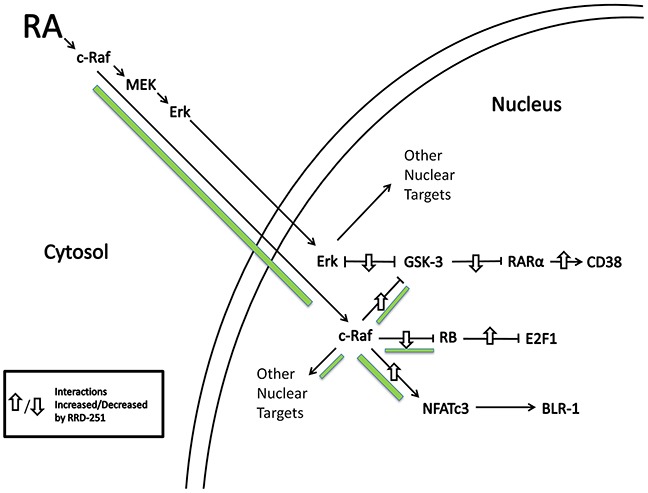
RA induces c-Raf nuclear translocation and binding to nuclear proteins RRD-251 augments those effects. Green accent shows flow of c-Raf. Up/down arrows show effects shown to be affected by adding RRD-251.

**Figure 9:**
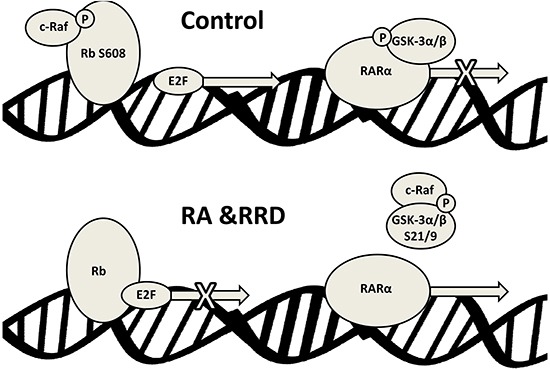
Schematic representation of the nuclear events induced by RA and RRD-251 co-treatment on RAREs

The present report shows that RRD-251 enhances RA-induced differentiation of HL-60 human myeloblastic leukemia cells, a patient derived NCI-60 reference cell line. RA and more so with RRD-251 causes nuclear translocation of c-Raf. The nuclear c-Raf potentially has various targets. Some is sequestered with pS608 RB and dissociates to increase availability to other targets as pS608 RB is lost with RA-induced cell cycle arrest. RRD-251 causes dissociation of c-Raf from RB increasing availability of nuclear c-Raf freed from RB sequestration. One c-Raf target is the NFATc3 transcription factor which enables activation of an upstream RARE by RA in the promoter of CXCR5; RA-induced early expression of which is necessary for cell differentiation to progress. RRD-251 increases RA-induced c-Raf binding to NFATc3. Another nuclear c-Raf target is GSK-3. c-Raf targeted GSK-3 has enhanced phosphorylation at the inhibitory site and dissociates from RARα in RA-treated cells, relieving RARα inhibition. RRD-251 promotes this by increasing nuclear c-Raf. As evidence of greater RRD-251 attributable RARα transcriptional activity, there is increased expression per cell of CD38, a receptor that drives differentiation. Thus we reveal a novel RA/c-Raf/GSK-3/RARα axis to be of importance for regulating differentiation (schematic diagrams, Figures 8 and 9).

It is perhaps worthy of note that the AML cells, HL-60, studied here are capable of bidirectional differentiation and that their granulocytic differentiation and monocytic differentiation are known to have molecular processes in common. Hence the general mechanism elucidated above for a signaling axis to RARα may be at work for VDR, too. Viability of this conjecture remains to be tested.

Medical management of AML patients remains difficult and outcomes are poor. In contrast APL, a subtype of AML, is highly responsive to RA and the remission rates are high. However, even with APL transient remissions with recurrence of resistant disease, as well as retinoic acid syndrome are sequela. Combination therapy including RA with another agent show promise, as we recently reviewed [3]. Enhancers of RA action offer the possibility of increasing RA efficacy and reducing dosages to mitigate toxicity. GSK-3 was demonstrated as an important modulator of RARα transcriptional activity and therefore a potential target for RA-induced differentiation therapy [8, 9]. Indeed, co-treatments of RA with inhibitors of GSK-3 have been shown to enhance the cellular response to RA in numerous AML lines beyond the APL subtype [8].

c-Raf, RB, and GSK-3, the principal molecular players in the present studies have a legacy in cell cycle/differentiation regulation. Traditionally, c-Raf, the protooncogenic homolog of v-Raf is known as a mitogenic and oncogenic signaling molecule that transmits signals from the plasma membrane to the cytoplasm and nucleus via the Raf/Mek/Erk signaling axis [16]. Transient activation of c-Raf is associated with proliferation and oncogenesis; however, a prolonged, durable activation and nuclear translocation is associated with RA-induced differentiation and cell cycle arrest [18]. Downstream of the signaling, the RB tumor suppressor protein regulates the cell cycle through interactions with cyclins, CDKs, and S-phase promoting factors, including the E2F family of transcription factors, chromatin and nucleosome remodeling complexes, and histone modifiers [20, 39]. As for other hematopoietic cell lines, during cell cycle progression of untreated HL-60 cells, RB is in the hyperphosphorylated state and is increasingly more phosphorylated going from G_1_ to G_2_/M; but begins being dephosphorylated in late G_2_ in RA-treated cells [20, 39]. Hypophosphorylated RB is only detectable in differentiating cells. Another downstream target of signaling, GSK-3 is an ubiquitously expressed target of insulin and growth factor signaling via PI3K and Akt which plays diverse roles in the regulation of metabolism, cell proliferation and apoptosis [40]. Having both oncogenic and tumor suppressive roles, GSK-3 and its dysregulation are implicated in numerous pathologies, where it may control transcription factors and transcriptional regulators. Recently, it was discovered that GSK-3 inhibition promotes RA-induced differentiation through regulating the transcriptional activity of RARα, a transcription factor driving RA-induced differentiation [8].

It has been previously shown that RRD-251 induces G_0_ arrest by dissociating c-Raf from RB, which sequesters and decreases expression of E2F. We find that RRD-251-induced dissociation of c-Raf from RB enhances c-Raf binding to NFATc3 and GSK-3 to enhance RA-induced differentiation and cell cycle arrest. RRD-251 increases pS9/21 inhibitory site phosphorylated GSK-3, decreases pY279/216 activating site phosphorylated GSK-3, and dissociates GSK-3 from RARα, to relieve its inhibition by GSK-3. Consequently RRD-251 causes an increase in the median cellular expression of CD38, a differentiation marker whose expression is dependent on RARα activity. Ectopic CD38 expression enhances MAPK signaling and RA-induced differentiation [28]. However, RRD-251 causes a transient reduction in RA-induced CD38 expression 8 hours post treatment that is no longer evident at 24 hours. The cause of this transient is undetermined, but suggests that RRD-251 short-circuited CD38 signaling to accelerate differentiation. This short-circuiting may reflect the enhanced availability of nuclear c-Raf after its dissociation from RB by RRD-251, thereby obviating the need for CD38 to enhance signaling. Together, these results indicate a putative RA/c-Raf/GSK-3/RARα axis that is susceptible to RRD-251 and represents a novel regulatory pathway for RA differentiation that may extend its utility beyond APL to other myeloid leukemias. Additionally RRD-251 may have further potential in combination therapies for leukemic differentiation utilizing 1,25-dihydroxy vitamin D3 as GSK-3 and RARα were recently found to regulate VDR expression and transcriptional activity [41-43].

## CONCLUSIONS

RRD-251 enhances RA-induced differentiation of human myeloblastic leukemia cells. RA-induced differentiation utilizes a novel RA/c-Raf/GSK-3/RARα axis that uses c-Raf mobilized to the nucleus. Some of that c-Raf is sequestered by RB, and RRD-251 dissociates it to enhance differentiation. This indicates the potential of combined RA and RRD-251differentiation therapy.

## MATERIALS AND METHODS

### Cell culture and treatments

Human myeloblastic leukemia cells (HL-60) were grown in a humidified atmosphere of 5% CO_2_ at 37°C and maintained in RPMI 1640 (Invitrogen, Carlsbad, CA) supplemented with 1% antibiotic/antimycotic (Sigma, St. Louis, MO) and 5% heat-inactivated fetal bovine serum (Hyclone, Logan, UT). The experimental cultures were initiated at a density of 0.2 × 10^6^ cells/mL for lysate collection at 24 and 48 hours post treatment. For lysate collected 72 hours after treatment and all flow cytometry experiments, cultures were initiated at a density of 0.1 × 10^6^ cells/mL. Cell growth and viability was monitored with hemocytometer and 0.2% trypan blue (Invitrogen, Carlsbad, CA) exclusion assay. All cells and lysate collected used the same treatment conditions of RRD-251 Hydrochloride (Sigma) (0 μM, 10 μM, or 20 μM) and RA (Sigma) (0 μM or 1 μM). Cells were collected 24, 48, or 72 hours post treatment. Three replicates of each experiment were performed unless otherwise noted.

### CD11b/CD38 expression studies by flow cytometry

HL-60 cells (0.5 × 10^6^) were harvested by centrifugation at 120 × g for 5 minutes. Pelleted cells were resuspended in 200 μL of PBS containing 2.5 μL of allophycocyanin-conjugated anti-CD11b antibody and phycoerythrin-conjugated anti-CD38 antibody (BD Biosciences). Following incubation for 1 hour at 37°C, cells were analyzed by flow cytometry (LSRII flow cytometer, BD Biosciences) using 633-nm red and 488-nm blue laser excitations. Gates to determine the percent of positive cells were set to exclude 95% of control cells. To determine relative CD38 expression per cell the median of the fluorescence histogram was derived, and shifts in relative median expression level with RRD-251 treatment were used to determine the effect on CD38 expression [44].

### Measurement of respiratory burst (inducible oxidative metabolism)

0.5 × 10^6^ HL-60 cells were harvested by centrifugation and resuspended in 200 μL of PBS containing 10 μM 5-(and-6)-chloromethyl-2′,7′-dichlorodihydrofluorescein diacetate acetyl ester (H2-DCF, Molecular Probes, Eugene, OR) and 0.4 μg/mL 12-O-tetradecanoylphorbol-13-acetate (TPA, Sigma) with incubation for 20 min in a humidified atmosphere of 5% CO2 at 37°C. Flow cytometric analysis was performed (BD LSRII flow cytometer) using 488-nm laser excitation and emission collected through 505 long-pass dichroic and 530/30 band-pass filters. The shift in fluorescence intensity in response to TPA was used to determine the percent of cells with the capability of generating inducible superoxide. Gates to determine the percent of positive cells were set to exclude 95% of control cells which had no TPA to induce the respiratory burst characteristic of differentiated mature cells. Cells that have not been RA-treated with or without TPA and RA-treated cells without TPA typically showed indistinguishable DCF fluorescence histograms [44].

### Cell cycle analysis

HL-60 cells (0.5 × 10^6^) were collected by centrifugation and resuspended in 200 μL cold hypotonic propidium iodide (PI) staining solution containing 50 μg/mL propidium iodine, 1 μL/mL Triton X-100 and 1 mg/mL sodium citrate. Cells were incubated overnight at 4°C and analyzed by flow cytometry (BD LSRII) using 488-nm excitation and collected through 550 long-pass dichroic and a 576/26 band-pass filters [44].

### Staining pS608 RB and DNA

1 × 10^6^ HL-60 cells were harvested by centrifugation at 700 rpm at room temperature for five minutes and washed with 500 μL cold PBS. Then, all samples were fixed by resuspending in 100 μL PBS with 2% paraformaldehyde and incubated at room temperature for 7 minutes, followed by the addition of 900 μL ice-cold methanol to yield a 90% methanol solution. Solutions were then incubated at −20°C for 1 hour. Samples were washed three times with cold PBS and resuspended in 200 μL of PBS or hypotonic PI containing 2.5 μL pS608 RB rabbit primary antibody. After incubation overnight at 4°C, samples were washed once with 1 mL cold PBS or PI hypotonic solution. Samples were then incubated for 1 hour at room temperature in 200 μL of PBS or hypotonic PI solution containing 2.5 μL FITC conjugated goat anti-rabbit antibody (BD Pharmingen). Samples were then washed twice and analyzed by flow cytometry (BD LSRII). Excitation was at 488 nm and emission was collected through a 530/30 band-pass filter. Fixed cells labeled with secondary antibody, but not primary antibody, were used to evaluate the background signal. Samples labeled with only FITC and only PI were used to correct for spectral overlap or “spillover.” The only fluorescence that showed spectral overlap was PI into FITC, which was corrected by a compensation of 2.0%. Three biological repeats were analyzed.

### Western blotting and immunoprecipitation

Cellular fractionation was accomplished with the NE-PER kit (Pierce) in accordance with the manufacturer's instructions with the addition of protease and phosphatase inhibitors, and all lysates were stored at −80°C until use. After lysate collection, cellular debris was pelleted by centrifugation at 16 060 × g for at least 10 minutes, and protein concentrations were quantified using the BCA assay (Pierce). Equal amounts of lysate were pre-cleared with PureProteome Protein G magnetic beads (Millipore, Billerica, MA, USA) for 2 hours, and then incubated overnight with beads and 1 μg of primary antibody. The beads were washed, boiled for 10 minutes, and dissociated proteins were resolved by SDS/PAGE analysis, followed by transfer to polyvinylidene fluoride (PVDF) membrane (Millipore). For western blotting, 25 μg of protein was resolved on a 7.5% polyacrylamide gel. The electrotransfer was for 1 hour at 400 mA. The membranes were blocked in milk for 1 hour before being incubated with the indicated primary antibody overnight at 4°C. Images were captured on a Molecular Imager VersaDoc MP 5000 System and analyzed using quantity one (Bio-Rad, Hercules, CA, USA). Antibodies used in Western blotting and immunoprecipitation were obtained from Cell Signaling (Danvers, MA) (RB, pS608 RB, pS780 RB, pS795 RB, pS807/811 RB, cdc6, TBP, GSK-3, RARα, RXRα, pS259c-Raf, c-Raf, p47^phox^), Santa Cruz (Dallas, TX) (IRF-1, Skp2, NFATc3), Invitrogen (Waltham, MA) (c-Raf, pS621 c-Raf), Novus Biologicals (Littleton, CO) (Sp1), and Abcam (Cambridge, UK) (pS608 RB). Each experiment was repeated three times, with a representative blot shown. Bar graphs show the averages from the independent repeats.

### Statistics

To determine statistical significance, one-way ANOVA tests were utilized with Bonferroni's multiple comparisons test comparing each sample against control, or RA. Significance was set at p<0.05. There were 3 repeats for each time point of CD38 (8, 24, 48 hours), CD11b (8, 24, 48, 72 hours), cell growth (24, 48, 72 hours) and ROS production (72 hours) and 6 repeats for cell cycle analysis.
